# Left subsuperior segmentectomy for a patient with early-stage lung cancer: a case report

**DOI:** 10.1186/s44215-022-00012-3

**Published:** 2022-11-15

**Authors:** Hodaka Oeda, Hidemi Suzuki, Taisuke Kaiho, Atsushi Hata, Ito Takamsa, Kazuhisa Tanaka, Yuichi Sakairi, Ichiro Yoshino

**Affiliations:** Department of General Thoracic Surgery, Chiba University Graduate School of Medicine, Chiba, 260-8670 Japan

**Keywords:** Subsuperior bronchus, Segmentectomy, Lung cancer, Surgery

## Abstract

**Background:**

The independent segment between the superior and basal segments is called the subsuperior segment (S*), which is rarely observed. We report a left S* segmentectomy in a patient with early-stage lung cancer.

**Case presentation:**

A 72-year-old man presented with a history of hilar cholangiocarcinoma. A left lung ground-glass nodule was detected during follow-up examination. The tumor shadow was localized in the left S* according to the findings of the three-dimensional image analysis system (SYNAPSE VINCENT®) with computed tomography-based analysis. S* segmentectomy was successfully performed with a sufficient surgical margin. The operation time was 147 min, and there was a small amount of bleeding. The pathological diagnosis was invasive adenocarcinoma measuring 3 mm. The tumor was successfully removed with a sufficient surgical margin. The patient was discharged from the hospital 8 days after surgery without any complications.

**Conclusions:**

S* segmentectomy is not typically performed in patients with lung cancer due to insufficient surgical margins. However, S* segmentectomy is a viable option for selected patients with pulmonary metastasis or early-stage lung cancer.

## Background

Previous studies have reported varying frequencies of the lung bronchi and vessel branching types [1–3]. The subsuperior segment (S*) is located between the superior (S6) and basal segments. Shimizu et al. reported the first video-assisted thoracic surgery (VATS) for anatomical right S* segmentectomy [4]. They also reviewed the pulmonary bronchovascular pattern variations using three-dimensional computed tomography (CT) angiography and the right lobe bronchography. Right S* was detected in approximately 20% of cases [5]. Other reports have focused on the anatomical thoracic abnormalities detected via three-dimensional CT [6, 7]. Maki et al. described the pulmonary vessels and bronchial anatomy of the left lower lung and found a S* in 24% of patients [8].

We report a case of early-stage lung cancer treated with left S* segmentectomy.

## Case presentation

**Fig. 1:**
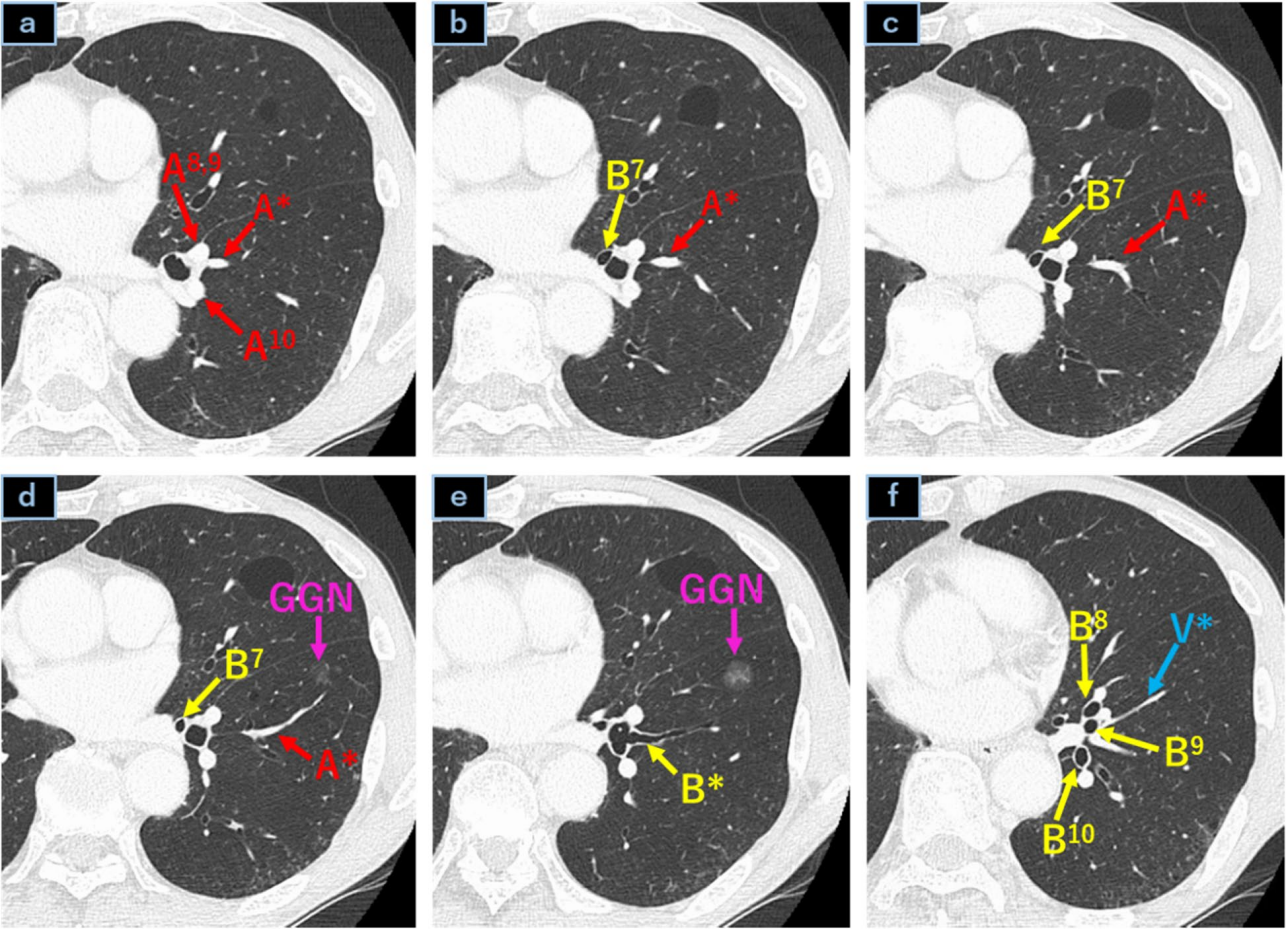
**a**–**f** Chest computed tomography images. A subsuperior segmental bronchus (B*) and B7 were detected by thin section computed tomography, and a ground-glass nodule (GGN) was detected in the subsuperior (S*) segment. The subsuperior segmental vein (V*) was included in the S*

A 72-year-old man presented with a history of hilar cholangiocarcinoma. A left lung ground-glass nodule (GGN) measuring 1.1 cm in diameter was detected in the left lung S* segment during follow-up examination (Fig. 1).

**Fig. 2:**
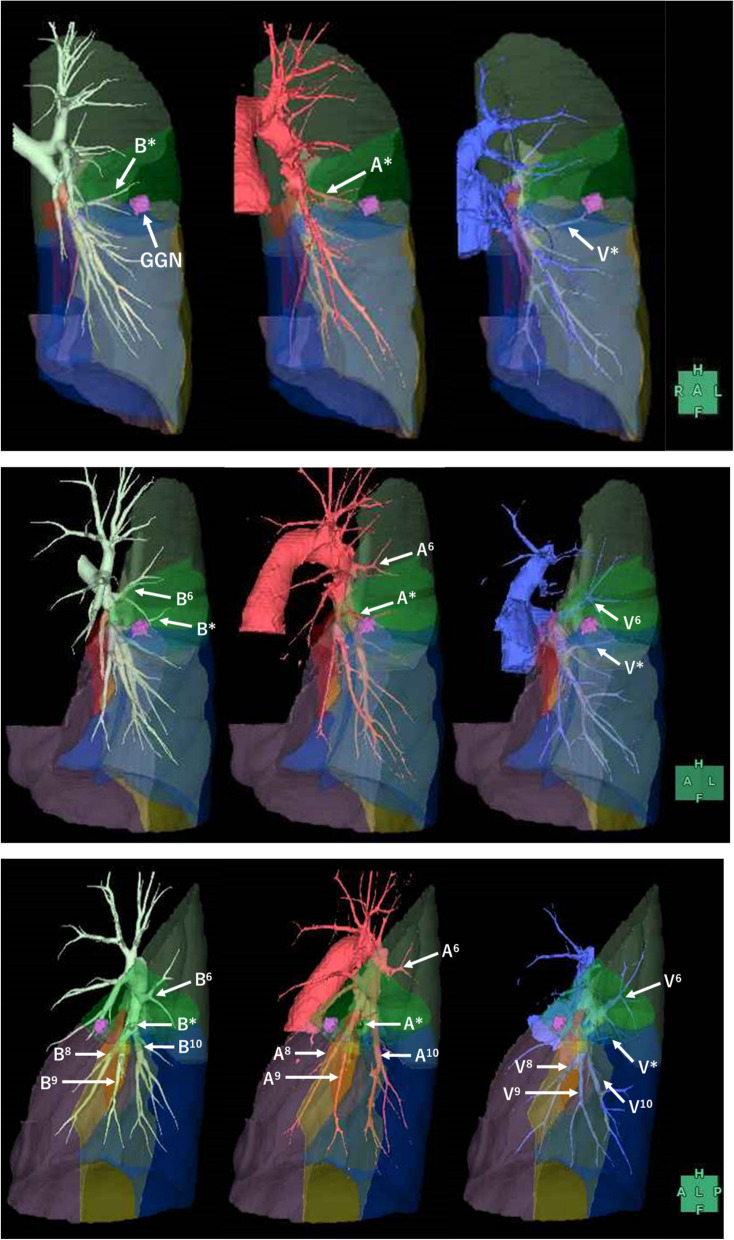
**a**–**c** A three-dimensional image finding constructed by SYNAPSE VINCENT. Relation to the lower bronchus, pulmonary artery, vein, segments, and tumor shadow. Colors of each segment and tumor: S*, green; S6, light green; S7, red; S8, purple; S9, yellow; S10, blue; tumor, pink. 2a Front view. 2b Between front- and left-side view. 2c Left-side view

Preoperative three-dimensional CT (SYNAPSE VINCENT® analysis) revealed an independent B* bronchus and pulmonary artery (A*) distinct from those of the S6 and basal segment (Fig. 2). In addition, both left B7 and B* were appreciated on CT (Fig. 1). The S* was surrounded by S6, S7, S8, S9, and S10 (Fig. 2). Since performing wedge resection was difficult, independent S* segmentectomy was considered a suitable operation for this case. The distance between the tumor and S8 was the closest, 4.5 mm, and we thought it necessary to be careful when resecting between segments.

**Fig. 3:**
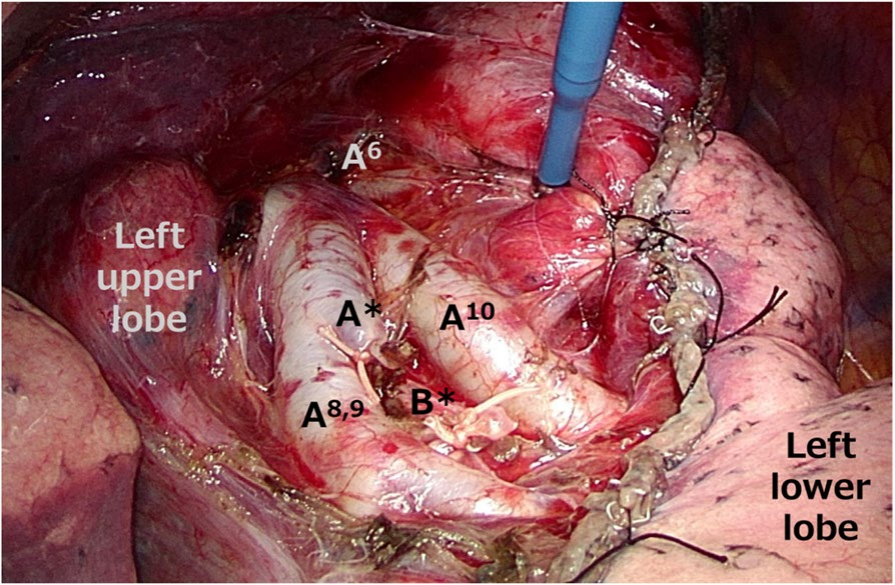
Thoracoscopic view. Structure of the segmental plane shown after S* segmentectomy in the operative view

First, the interlobar fissure was divided to identify the pulmonary artery. After identifying the superior segmental artery (A6), the peripheral side of the pulmonary artery was dissected. A* was identified between the basal segmental arteries A8 + 9 and A10. Second, after ligating and dissecting A*, the accompanying B* was identified. The tumor was palpable from the pleural surface, and a small-diameter bronchoscope was inserted into the orifice of B*, and selective air injection was performed. The S* segment was expanded until an inflation-deflation line was apparent. Finally, after marking the inflation-deflation line with conventional electrocautery, the central part of the intersegmental plane was dissected with electrocautery, and the peripheral part of the intersegmental plane was dissected along the marking line with automatic sutures (Fig. 3). The shortest margin distance from the tumor was S8/S*; we paid special attention to secure surgical margin dividing between S8 and S* with a sufficient surgical margin > 2 cm.

The operation time was 147 min, and there was a small amount of bleeding. The pathological diagnosis was invasive adenocarcinoma measuring 3 mm. The tumor was successfully removed with sufficient surgical margins. The patient was discharged from the hospital 8 days after surgery without any complications.

## Discussions and conclusions

Recent studies have reported the frequencies of right and left S* to be 20% [5] and 24% [8], respectively. An S* has an independent subsuperior segmental bronchus (B*) and pulmonary artery (A*), which has a small volume. S* is rarely observed, and segmentectomy is not typically performed in patients with primary lung cancer due to insufficient surgical margins. However, S* segmentectomy was deemed appropriate for early-stage lung cancer patients with pure GGN, metastatic lung tumor, and nonmalignant diseases, such as granulomas and congenital bronchial atresia, located in a part unresectable by wedge resection.

Identification of the intersegmental plane is one of the essential techniques for pulmonary segmentectomy [4, 5]. Since this left S* segmentectomy has multiple segmental planes between the S6 and basal segment, selective ventilation via bronchoscopy is effective and useful. We previously reported near-infrared-guided pulmonary segmentectomy after endobronchial indocyanine green (ICG) injection [9]. ICG injection via the bronchus or intravenous technique is useful in S* segmentectomy. We performed a left S* segmentectomy via a small open thoracotomy with a thoracoscope. Less invasive approaches such as VATS or uniportal VATS are technically possible [4, 10]. Even robot-assisted segmentectomy can be considered because firefly fluorescence imaging with the da Vinci Surgical System to utilize ICG is available [11]. In this case, we preoperatively identified an independent B* bronchus and pulmonary artery (A*) distinct from the S6 and basal segment. The left S* segmentectomy was safely performed. Furthermore, left B7, seen in 7.9% of cases [8], was also detected. Failure to note these anatomic variants preoperatively could have led to an inadvertent left S8 segmentectomy. Preoperative three-dimensional multi-dissector CT angiography allows visualization of the pulmonary vasculature and bronchi anatomy to help detect anatomical variants. This was recommended for surgical planning in patients undergoing lung resection, particularly in cases with complicated segmentectomy for lung cancer [4, 11]. For non-small cell lung cancer with a major axis of 2 cm or less and a lesion-filled area ratio of greater than 0.5 to the maximum lesion diameter, segmental resection should be standard surgery [12]. Although the lesions in this case were relatively peripheral, it should be considered that this may not be the case for more centrally located tumors or larger tumors, in which multi-segment resection is preferred. Left S* segmentectomy is technically feasible for selected patients with nodules in the S*. Three-dimensional CT findings are useful for preoperative planning for S* segmentectomy.

## Data Availability

All data supporting the conclusions of this article are included within the published article.

## Abbreviations

S*: Subsuperior segment
VATS: Video-assisted thoracic surgery
GGN: Ground-glass nodule
CT: Computed tomography
ICG: Indocyanine green
